# Influence of the Incubator as Direct Patient Environment on Bacterial Colonization of Neonates

**DOI:** 10.3390/microorganisms9122533

**Published:** 2021-12-07

**Authors:** Isabel Lange, Birgit Edel, Kristin Dawczynski, Hans Proquitté, Mathias W. Pletz, Frank Kipp, Claudia Stein

**Affiliations:** 1 Institute for Infectious Diseases and Infection Control, Jena University Hospital, Am Klinikum 1, 07747 Jena, Germany; Mathias.Pletz@med.uni-jena.de (M.W.P.); Frank.Kipp@med.uni-jena.de (F.K.); Claudia.Stein@med.uni-jena.de (C.S.); 2 Institute of Medical Microbiology, Jena University Hospital, Am Klinikum 1, 07747 Jena, Germany; Birgit.Edel@med.uni-jena.de; 3 Department of Pediatrics Weimar, Sophien-und Hufeland Klinikum, Henry-van-de-Velde-Str. 1, 99425 Weimar, Germany; K.Dawczynski@klinikum-weimar.de; 4 Unit of Neonatology, Department of Paediatrics, Jena University Hospital, Friedrich Schiller University Jena, 07747 Jena, Germany; Hans.Proquitte@med.uni-jena.de

**Keywords:** healthcare-associated infections, colonization, neonatal care, incubator, whole genome sequencing, infection control, transmission, patient environment

## Abstract

Background: Preventing healthcare-associated infections (HAI) in neonatal intensive care units is a challenge of highest priority. For further insight into the incubator as direct patient environment and potential source for contamination, we present data correlating microbiological samples of very low birthweight infants in the form of colonization results of surveillance screenings with samples of their associated incubator in this study. Methods: Samples were taken via rectal and throat swabs of neonates as well as Polywipe^®^ sponges for the incubator. If the same bacterial species was found in corresponding neonate and incubator samples, whole genome sequencing via Illumina technology was performed. Results: 52 microbiological species matches were found, and 30 matches were sequenced where we found 26 clonal pairs (12 *E. faecalis*, 10 *S. aureus*, 2 *E. coli*, 1 *E. cloacae*, and 1 *E. faecium*). Conclusion: The combinations of measurements of weekly screenings swabs, probing of surfaces with Polywipes^®^, and whole genome sequencing showed transmissions of microorganism and risk for potential non-physiological colonization of neonatal infants.

## 1. Introduction

Preventing infections in neonatal intensive care units is a high-priority challenge, especially for infants with very low and extremely low birth weight (<1500 g, VLBW, <1000 g, ELBW).

After birth, the process of physiological colonization for neonates depends on delivery mode, first maternal contact, and the surrounding environment [1,2,3]. Certain pre- and probiotics are given naturally to the neonates via bacterial sources through maternal microbiotas via translocation (intestinal) or dissemination (blood stream) through in utero pathways of amniotic fluid and the placenta. Contact with vaginal and rectal maternal microbiota through vaginal delivery represents another physiological colonization process of the gut microbiome of neonates [4]. An impending problem for the patient group at risk are healthcare-associated infections (HAIs), due to either Gram-negative or Gram-positive organisms [5,6]. 

In Germany, the commission for hospital hygiene and infection control (Kommission für Krankenhaushygiene und Infektionsprävention, KRINKO) recommends a weekly screening for bacterial microorganisms via throat and rectal swab of neonates with VLBW in neonatal intensive care units (NICU) as a measure to prevent healthcare-associated infections, especially those with multi-resistant microorganisms [7,8,9]. Examples of bacteria which act as potential pathological threats include multi-resistant organisms, species that lead to invasive infections (*Acinetobacter* spp., *Klebsiella pneumoniae* and *Staphylococcus aureus*), as well as species that have a special pathogenicity and high potential to be involved in nosocomial outbreaks (*Enterobacter* spp., *Serratia marcescens*, and *Pseudomonas aeruginosa*). 

Colonization of neonates is of special interest since their immune system is compromised and colonization’s potentially lead to infections in this unique cohort of patients [10,11]. Colonization is an inevitable and normal process influenced by getting in contact with both the parents and medical staff. Brooks et al. described how the colonization of parents and neonates shape the microbiome of NICU rooms and how the specific microbiome is still detectable despite regular cleaning [12].

*S. aureus*, *Enterobacteriacae*, and *Enterococcaceae* often have been described in correlation with infections and bacteremia in neonates as well as severe outbreaks [13,14,15,16,17]. In a study by Akinboyo et al., all neonates from their level IV NICU were screened over two years, revealing 13% of the infants to be colonized with *S. aureus* [18]. In Germany, there is a surveillance system implemented for nosocomial infection surveillance for preterm infants in neonatal departments and ICUs (called NEO-KISS). It collects and includes data about cases with pneumonia, necrotizing enterocolitis, and sepsis, as well as the microbiological species that are associated with the diagnosis. Pneumonia and sepsis with *S. aureus* and *E. faecalis* are two of the top five species related to these infections [19]. The incubator represents a potential source for nosocomial contaminations because, in Germany, all VLBW neonates are handled in an incubator, which is in use for approximately fourteen days before disinfection and turnover.

In this study, we present data reflecting the direct patient environment of VLBW infants in the form of microbiological probing of the attributive incubator, correlated with colonization results from the surveillance screening. These positive species were then analyzed via whole genome sequencing (WGS) in order to generate transmissions and colonization pathways. Subsequently, conclusions could be drawn to improve hygiene measures for preventing infections in VLBW infants.

## 2. Materials and Methods

### 2.1. Incubator Disinfection and Sampling

From January 2019 to August 2020, we analyzed all microbiological screenings from neonates via rectal or throat swab in our neonatal intensive care unit (NICU level IV with 27 beds). Whenever the incubators were replaced for suitable disinfection, we sampled the incubator that the corresponding VLBW was located in. The two incubator models in use are as follows: Isolette^®^ C2000 (Hill-Rom Inc., Air Shields^®^, Hatboro, PA, USA) and Giraffe^®^ Incubator (GE Healthcare, Laurel, MD, USA). The disinfection process was completed no later than every ten days for the Isolette^®^ model and every 14 days for the Giraffe^®^ model. In the meantime, the neonates were taken out for kangarooing time with their parents or in some cases transferred to another hospital. The incubators were dismantled of all demountable parts and soaked or wiped off with Dismozon^®^ plus 0.4% (Hartmann AG, Heidenheim, Germany). An application time of 60 min was needed. After the application time, the empty water supply piece, water bottle, and water hose were sent to be sterilized, and the acrylic glass hood was cleaned with fresh water and polished. All parts were reassembled, dried, and aired out for 24 h with the incubator turned on to ensure that no residue moisture or smells were left. Before being used again, the suitable disinfection process date was documented. 

**Figure 1:**
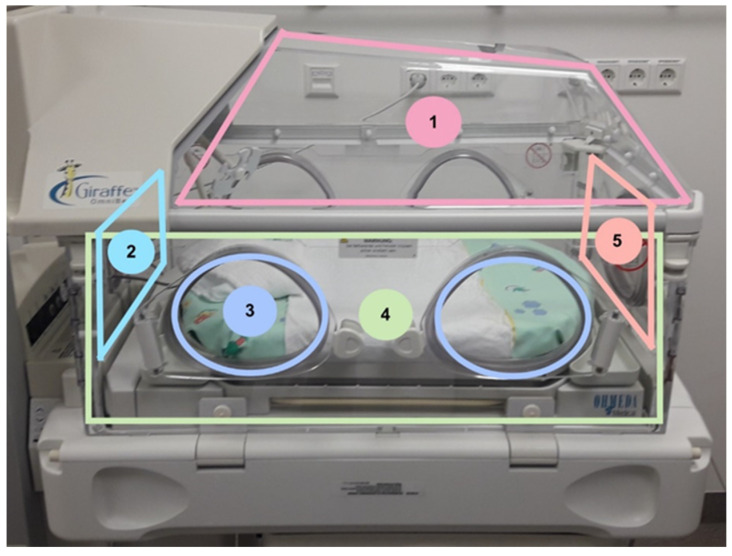
Sampling location of the incubators. All samples were taken from different areas inside of the incubator including the lid (1), one side (2 and 5), door (3), headpiece and base (4).

From every incubator, we gathered ten samples with two different sampling methods of different areas inside for microbiological testing. When an incubator was up for disinfection, hygiene-specialized nurses were informed by the NICU staff and carried out the sampling. Five samples were taken with replicate organism detection and counting (RODAC™) contact plates (Thermo Fisher, Wesel, Germany), and the same five locations were wiped with premoistened Polywipes^®^ sponges (Medical Wire, Corsham, Wiltshire, UK). Sampling with RODAC™ plates was performed via a stamp technique of the sheep blood agar on the area of the locations listed in Figure 1. Polywipes^®^ sponges were buffered wipes which could take up microbes from a larger area than swabs or contact plates. With a sterile glove, the wipe was removed from the sample package, and the surface to be tested was wiped off. After removal, the wipe was placed back in the sterile transport box. The areas are marked in Figure 1. Overall, 62 incubators were included in this study and we focused on areas inside of the incubator. The locations were chosen, so all inside surfaces of the incubator are representative.

### 2.2. Neonatal Screening and Match Process

Neonates received an initial throat and rectal swab after birth or when shifted to the hospital from nearby clinics. During the following days of admission, a weekly microbiological colonization screening via throat and rectal swab was performed after the recommendations of the German KRINKO [7]. All microbiological results were gathered in a spreadsheet. To be included in the study, the neonates and incubator samples were matched for pathological microorganisms during the 10–14-day period until the incubator was disinfected. Whenever the same species was found in one of the ten incubator samples and in the weekly screening results of the patient, the isolate of the incubator sample and the one of the neonatal screening were analyzed via WGS. A match process involved finding the same species and requesting the isolates for WGS. Clonality of the bacterial isolates was determined to show potential transmissions and provide input regarding the colonization of the patients. 

### 2.3. Enrichment and Incubation of the Incubator and the Patient Samples

To enrich the bacteria within the Polywipes^®^, 50 mL of CASO broth containing TLHC (tryptic soil broth with neutralizers, Merck KGaA, Darmstadt, Germany) was added to each cloth. The contained detergents (3% tween, 0.3% lecithin, 0.1% histidine, and 0.1% cysteine) inactivate any present disinfectants. The broth was incubated at 37 °C for 24 h. Then, 100 µL of the broth was streaked onto Columbia blood agar (with 5% sheep blood, Becton Dickinson, Franklin Lakes, NJ, USA) and Drigalski lactose agar (OXOID Deutschland GmbH, Wesel, Germany). The plates were incubated at 37 °C for 48 h.

The rectal and pharyngeal swabs from the neonates were streaked onto Columbia blood agar (with 5% sheep blood, Becton Dickinson, USA) and Drigalski-lactose agar (OXOID Deutschland GmbH, Germany). The plates were incubated at 37 °C for 48 h. 

All relevant colonies from wipes and swabs were isolated, identified with MALDI-TOF (Vitek MS, BioMerieux Deutschland, Nuertingen, Germany), and sampled for sequencing.

### 2.4. DNA Isolation and Whole Genome Sequencing

High-throughput WGS and subsequent data analysis were performed using a MiSeq instrument (Illumina, San Diego, CA, USA), as recently described [19]. For the library preparation, we used the Nextera DNA Flex Kit and Nextera DNA CS Indexes (Illumina, San Diego, CA, USA). Genomic DNA was paired-end sequenced with a MiSeq Reagent kit v2 250 bp (Illumina) with an average insertion size of 300 bp. The resulting reads were quality-trimmed and de novo assembled using the Velvet algorithm integrated in the Ridom Seqsphere^+^ software version 7 (Ridom GmbH, Muenster, Germany). For core genome multi-locus sequence typing (cgMLST), we used species-specific public cgMLST scheme for a gene-by-gene comparison on an allelic level [19,20,21]. To illustrate the clonal relationships between different isolates, minimum-spanning tree analyses were performed based on the determined allelic profiles using the Ridom Seqsphere^+^ software with the parameter “pairwise ignore missing values”. We defined a clonal transmission event if the isolates differing ≤6 alleles for *S. aureus*, ≤10 alleles for *E. coli*, and ≤5 alleles for *E. faecalis* [19,22]. This whole genome shotgun project was deposited at DDBJ/ENA/GenBank under the accession JAINWQ000000000-JAINZL000000000 and JAIOGN000000000-JAIOGP000000000. The version described in this paper is version PRJNA759098.

## 3. Results

### 3.1. Patient Population and Microbial Colonization

Sixty-two neonatal patients were included in this study. Gestational ages ranged from 23 to 34 weeks (median and average of 28 weeks) and a weight range from 395 g to 3410 g (median 977 g, average ± standard deviation 1076 ± 483 g). Furthermore, 20 of the 62 neonates were VLBW infants, while 34 were ELBW infants. 

For all 62 neonates, rectal and throat swabs were analyzed. Further results from eye swabs, tracheal secretions, or stool samples were not considered because they did not provide other germs than the rectal and throat screening swabs. We did not include certain factors, such as the delivery mode or feeding methods in this study. 

In throat swabs, we found 16 positives for *S. aureus*; 5 for *E. coli*; as well as other occasional finds, such as for *R. ornithinolytica*, *E. faecalis*, *E. cloacae*, *M. morganii*, *K. pneumoniae*, *A. baumannii*, and *C. freundii*. 

In rectal swabs, we found 26 *E. faecalis*, 15 *S. aureus*, 14 *E. coli*, 8 *E. cloacae*, and 5 *K. pneumoniae*. Occasionally, we also found two *E. faecium*, *R. ornithinolytica*, and *C. freundii*, and one of each of *K. oxytoca*, *C. cruseii*, *S. parasanguinis*, *S. hominis*, *S. oralis*, and *M. morganii*.

### 3.2. Microbial Findings of Neonatal Incubators

**Figure 2:**
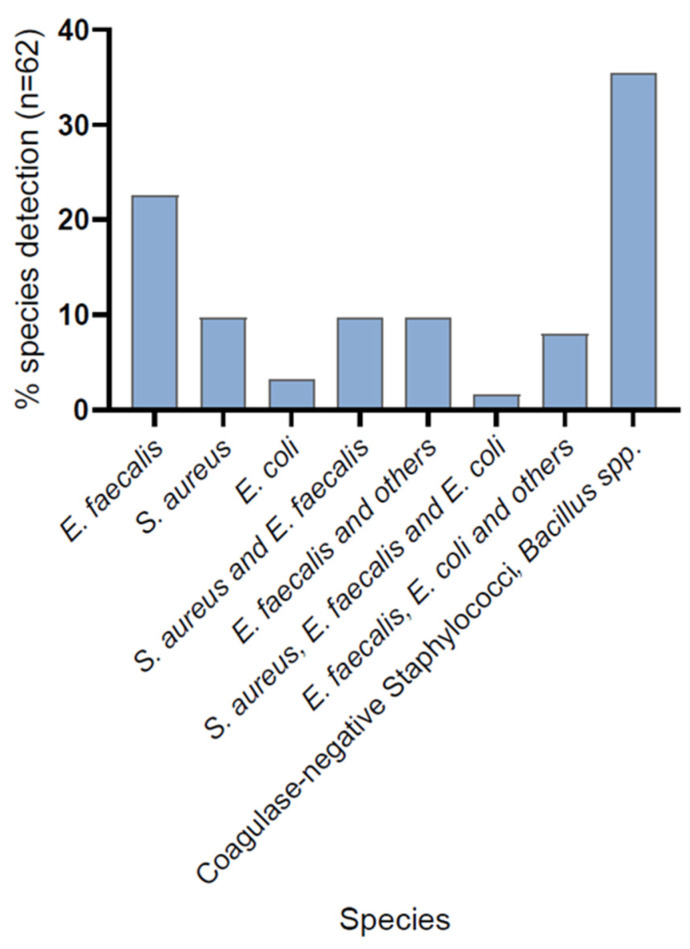
Bacterial species found in the 62 incubators. Distribution of potential pathologic microorganisms found on incubators with Polywipe^®^ sponges.

Of all 62 incubators sampled with RODAC (five per incubator) and Polywipes^®^ (five per incubator), we found coagulase-negative staphylococci (CoNS) and *Bacillus* spp. microbes in 22 incubators. In the other 40 incubators, we found either *E. faecalis*, *S. aureus*, *E. coli*, or more than one of those species on at least one of the five sampled locations. Fourteen incubators had only *E. faecalis*, six *S. aureus*, and two had only *E. coli*. In six incubators, we found *E. faecalis* and other Gram-negative microorganisms, such as *K. oxytoca* or *E. cloacae*. Furthermore, six had *S. aureus* and *E. faecalis*, and five had *E. faecalis* and *E. coli*. In one incubator, we detected three species in the Polywipes^®^ samples, which were *S. aureus*, *E. coli*, and *E. faecalis* (Figure 2). 

Parallel to the neonatal incubator detections, we compared two preanalytical detection strategies where we used two different testing materials for the same incubator location. Excluded from these findings of pathological species were CoNS and *Bacillus* spp. With Rodac plates, we only found potential pathologic microorganisms (*E. coli*, *E. faecalis*, *S. aureus*, *K. oxytoca*, *E. cloacae*, and *C. freundii*) in 14.5% of the analyzed samples (9/62). With Polywipes^®^, we detected pathologic microorganisms in 64.5% of all tested incubators (40/62). The two microbial test procedures differ in a quantitatively significantly way (McNemar test *p* = 10^−8^). Therefore, with Polywipes^®^, we found that microbes appeared four times more often than with RODAC plates. 

### 3.3. Bacterial Species Comparison between Neonatal and Their Associated Incubator

Overall, we found matching species in the incubator and the corresponding neonatal screening sample in 52 cases (38/62 neonate and incubator pairs, plus 14 pairs which result in the same neonate with their incubator being colonized with more than one species). The species we found in most matches were *E. faecalis* (*n* = 23) and *S. aureus* (*n* = 13). All *S. aureus* were methicillin-sensitive (MSSA) and all *E. faecalis* were vancomycin-sensitive. Furthermore, there were seven *E. coli* matches; three *E. cloacae*; two *E. faecium*; and one each of *K. oxytoca*, *K. pneumoniae*, *C. freundii*, and *R. ornithinolytica*. All of these Gram-negative findings were sensitive to beta-lactam and fluorchinolone. The two cases of *E. faecium* were also sensitive to vancomycin. No species match was found in 24 out of 62 neonate and incubator pairs. In some cases, more than one species matched within the neonate and incubator pair.

### 3.4. Clonality and Cluster Analysis

**Figure 3:**
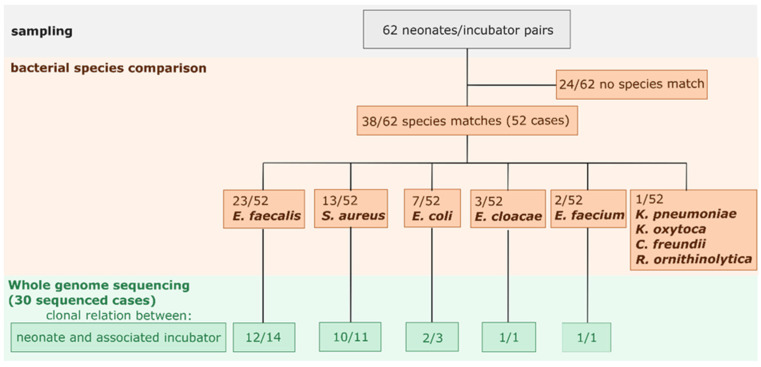
Flowchart of the sampling, species comparison, and sequencing.

We identified 74 isolates for *E. faecalis* and *S. aureus*. There were 17 rectal *E. faecalis* isolates and 21 *E. faecalis* incubator isolates (14/23 neonate and incubator pairs) which were included in the whole genome sequencing analysis. For *S. aureus*, we received 24 neonatal screening isolates and 12 incubator isolates (11/13 neonate and incubator pairs). Other species isolates analyzed via whole genome sequencing were eight *E. coli* (3/7 pairs neonate/own incubator), three *E. cloacae* (1/3 pairs neonate/own incubator), and two *E. faecium* (1/2 pairs neonate/own incubator). The results are summarized in Figure 3. 

The missing 18 pairs, which exhibited a species match, were not sequenced because of logistical issues, where we did not receive any of the pairs or, in some other cases, we only received either the patient or only the incubator isolate.

#### 3.4.1. Clonal Comparison: Neonate and the Corresponding Incubator for *E. faecalis*

**Figure 4:**
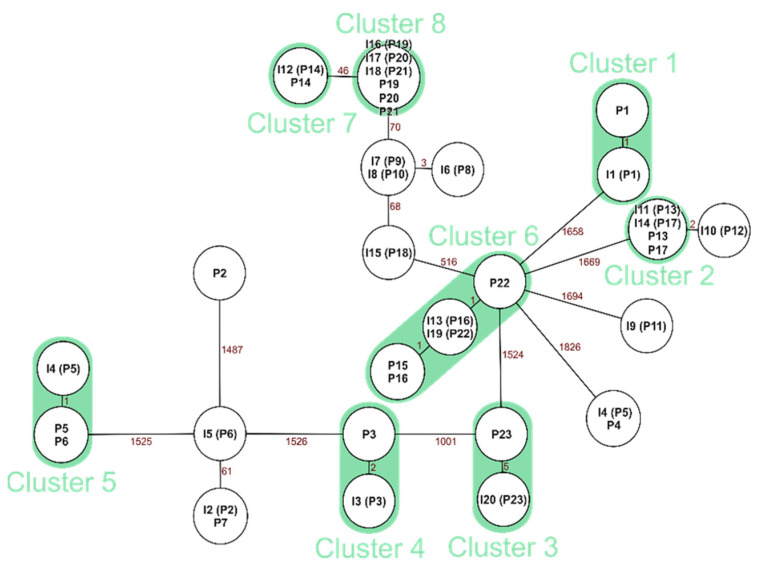
Minimal spanning tree of *E. faecalis* with marked clonal neonate and incubator pairs. P = the patient isolate, I = the incubator isolate. Isolate numbers in parenthesis show the patient isolates that the incubator isolate belongs to. Green marks pairs of neonates and their own incubator. If there is no allelic difference between the isolates, isolates are listed in one circle. The numbers between isolates display the amount of allelic differences.

For *E. faecalis*, we found 12 clonal pairs of the 14 sequenced neonatal patient and incubator pairs (Figure 4). 

#### 3.4.2. Clonal Comparison: Neonate and the Corresponding Incubator for *S. aureus*

**Figure 5:**
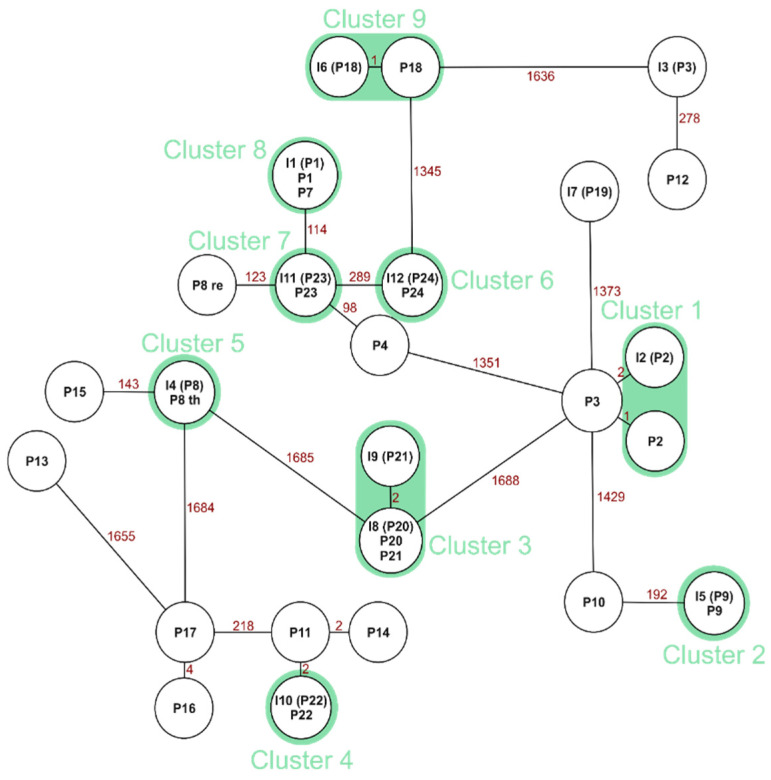
Minimal spanning tree of *S. aureus* with marked clonal neonate and incubator pairs. P = patient isolate, I = incubator isolate. Isolate numbers in parenthesis are showing the patient isolates the incubator isolate belongs to. Green marks pairs of neonates and their own incubator. If there is no allelic difference between the isolates, isolates are listed in one circle. Numbers between isolates display the amount of allelic differences.

For *S. aureus*, we found ten clonal pairs out of 11 pairs of neonate and incubator samples from which whole genome sequencing could be performed (Figure 5).

Of the seven *E. coli* matches, we received three of them for WGS analysis. Furthermore, we analyzed one *E. cloacae* pair and one *E. faecium*. We additionally found two of the three pairs of *E. coli* to be clonal as well as the *E. cloacae* and *E. faecium* pair between neonate and incubator.

In summary, out of the 52 microbiological matches for all species between neonate and their incubator, we sequenced 30 neonate incubator pairs and were able to display clonal pairs via whole genome sequencing in 26 cases (12 *E. faecalis*, 10 *S. aureus*, 2 *E. coli*, 1 *E. cloacae*, and 1 *E. faecium*).

## 4. Discussion

In this study, we examined throat and rectal swabs of 62 neonates to receive information about colonization as a preventive measure for infections. These swabs were carried out weekly at our NICU for potential pathologic bacteria. The neonatal colonization is physiological but differs between preterm and full-term infants [23]; hence, colonization screenings are used to control potential infectious developments and spreads in preterm infants, especially in ELBW. Furthermore, finding the same pathogen in epidemiological contexts presents important information about pathogen spreading in neonatal wards. The colonization process after birth is a topic of many studies, often regarding the whole microbiome of neonates [24]. The information gathered in this study about colonization of our neonates is not comparable to the analysis of neonatal microbiomes because we selectively cultivated our samples. We also did not consider the delivery or feeding mode. Nevertheless, the microbiological findings show typical, potential pathological bacteria that can lead to HAI. 

The colonization of neonates is known to be influenced by their environment [24]. The incubator that a neonate lies in is the nearest patient environment and presents a potential for transmissions of pathogens since contact is necessary for medical and tending procedures. Every ten to fourteen days, incubators in use were changed for suitable disinfection in our NICU. Before the disinfection process began, infection control nurses took samples from five different locations on the inside of the incubator for microbiological diagnostics. We were able to collect 38 species matches between the neonate and their own incubator isolate (52 cases). From these 52 cases, we included 30 pairs for whole genome sequencing to determine the clonal relationship. Overall, 26 clonal relations were revealed. As a result, we were able to prove a high degree of relationship between the colonization isolates of the neonates and the isolates of their own incubators, especially for the species *S. aureus*, *E. faecalis*, and *E. coli*. In 22 out of 62 incubators, we found CoNS which can play a role in bacteraemia cases in neonates [25]. Similar pathogens have also been found on incubators in a study by Chavignon et al. [26]. 

The materials used to detect the pathogens within the incubator were RODAC plates and Polywipe^®^ sponges. Regarding the results, Polywipes^®^ detected more pathogens than RODAC plates. We found that Polywipe^®^ sponges are more suitable for microbial detection in hospital environments. Our results suggest that Polywipes^®^ detect more potential pathological microorganisms than the stamp technique with RODAC plates. The wipes are very well suited to test larger surfaces, as they absorb more material and have a larger surface than simple swabs. In another study, we used Polywipe^®^ sponges to detect multi-resistant bacteria in different patient environments, which had similar positive results within the diagnostic yield of pathogens [27]. However, it should be noted that the RODAC plates can be used for quantification, which is not possible with the Polywipe^®^ sponges. The RODAC plates are better suited for checking final disinfection, for example. With the Polywipes^®^, you only have a qualitative statement: is the species present or not. This method is suitable to prove the presence of specific organisms or resistances resulting from high recovery and a large sampling area.

The microbiological findings of the same species in the throat and rectal screening and inside the incubator alone does not provide sufficient information, if transmission of the same bacteria coincidentally occurred. Therefore, we added the WGS for clonality analysis to receive further information about potential nosocomial transmissions. To our knowledge, this has not been carried out in previous studies. 

Clonal relations between a neonate and their own incubator can be expected since they are in direct contact to the inside of the incubator. In this study, we found 12 out of 14 neonate incubator matches for *E. faecalis* to be clonal (85%) and 10 out of 11 *S. aureus* matches (90%). It remains unclear, however, if the bacteria colonization occurred first on the incubator or on the neonatal patient. Brooks et al. found that “a component of premature infant gut colonization is the cycle of microbial exchange between the room and the occupant” [23], whereas the systematic review of Hartz et al. found that “literature revealed that various aspects of living within the NICU environment do influence the microbiome of infants” [28]. For the results of *E. faecalis*, epidemiological connections overall were scarce, and no incubators were shared between the clonal matches. 

In our study, there is evidence that the incubator has an essential impact on the colonization of neonates and that a high number of neonates are colonized with clonal isolates. A potential improvement and intensification of disinfection processes of the incubators is needed. More studies are needed to analyze disinfection measures of incubators. An option to consider is the use of UV light [29,30] as an additional disinfection procedure while the incubator is in use, i.e., whilst the neonate is kangarooing. Through the intensification of measures, transmissions could be interrupted or prevented. 

Additional preventive measures of nosocomial colonization and infections in neonates implement various infection control measures, in turn encouraging the use of breast milk, and decreasing the empirical use of antibiotics. To prevent further outbreaks of the colonization and infection of multi-drug-resistant organisms in NICUs, Cipolla et al. propose the following measures: performing hand hygiene, cohorting and isolating patients, screening healthcare workers, and performing admission and periodic surveillance cultures [28,31,32].

## 5. Conclusions

The combinations of measurements of weekly screenings via throat and rectal swabs, probing of surfaces with Polywipes^®^, and whole genome sequencing showed the transmission of microorganism and risk for potential non-physiological colonization of neonatal infants. These transmissions need to be diminished to prevent infections in the high-risk group of neonates with VLBW.

## Data Availability

All relevant data are within this article.
